# Self-Management in Daily Life with Psoriasis: An Integrative Review of Patient Needs for Structured Education

**DOI:** 10.1155/2012/890860

**Published:** 2012-12-06

**Authors:** Gitte Susanne Rasmussen, Helle Terkildsen Maindal, Kirsten Lomborg

**Affiliations:** ^1^Department of Dermatology, Aarhus University Hospital, 8000 Aarhus, Denmark; ^2^Department of Public Health, Aarhus University, 8000 Aarhus, Denmark

## Abstract

The aim of this integrative review is to identify and discuss patient needs for education to support self-management in daily life with psoriasis. As psoriasis increasingly gains recognition as a serious chronic autoimmune skin disease with long-term impairment on the life course, and not mainly a cosmetic problem, nurses are highly challenged to develop efficient education to support patient self-management. The paper includes five stages: (1) problem identification, (2) literature search, (3) data evaluation, (4) data analysis and synthesis, and (5) presentation, based on theoretic scaffolding around the concept “need.” Nineteen of 164 original papers within nursing, medicine and psychology, and reflecting patient perspective were included. To capture the patients' cultural understanding of the implications of the disease and care, we developed an interlevel model indicating that self-experienced burden of disease and its visibility, personal conditions such as illness perception, and the patient's age at onset time are high-impact factors that should be addressed in future structured patient education programmes. The research on patient needs has hitherto focused on adults, but the problems and vulnerability associated with having a chronic and visible disease during adolescence must be acknowledged, and patient education initiatives designed for this young group are recommended.

## 1. Introduction

Psoriasis is a common chronic inflammatory immune-mediated skin disease characterised by red, thickened, and scaly skin. Approximately 2 per cent of the world's population currently live with psoriasis, the prevalence appearing to be highest in Scandinavian countries and Northern Europe [1]. Recent research reports that the onset of the disease occurs before the age of 16 in 27–45% of the cases and before the age of 20 in more than 50% of the cases [2, 3]. The course of disease varies greatly, and treatment is often time-consuming and distressing [4].

The growing interest in psoriasis has underlined that the disease constitutes a substantial physical burden and may impact the patient's life course in various ways. Numerous studies in the field of Health-Related Quality of Life have quantified the impact of psoriasis on daily functions and activities [5–8] as well as quality of life to an extent similar to other major diseases such as cancer, diabetes, and cardiovascular disease [9]. It is commonly recognised that the stigma associated with visible skin lesions may lead to psychosocial adjustment problems [10, 11].

Psoriasis is not only a cosmetic problem for the patients. A recent review has reported an association between psoriasis and severe physical conditions such as psoriatic arthritis, cardiovascular disease, metabolic syndrome, and Crohn's disease. In addition, life expectancy is reduced by several years in individuals with severe disease, mainly caused by increased risk of heart disease [12].

This new insight has resulted in an upcoming shift in the treatment of psoriasis, from exclusively treating a skin disease towards comprehensive disease management including early diagnosing, monitoring, and intervention. This shift has increased the focus on developing new interventions, and patient education is one of several initiatives that aim to motivate people to chronic disease self-management. Self-management is generally defined as the day-to-day activity that an individual carries out to manage his/her long-term health condition. There is no gold standard definition of self-management within nursing science. The on-going discussion to clarify and develop this concept [13–18] emphasizes that self-management can be viewed from three perspectives: a process reflecting the patients perspective; a structure that reflects the perspective of the health professionals often described as educational activities; and a goal or an outcome for the patient, for example, to improve quality of life or maintain health or well-being. In accordance with the aim of this review it seems relevant to adopt the patient's perspective on the concept of self-management. The concept refers to the activities people undertake to create order, discipline and control in their lives. Self-management is an active and dynamic process of learning, trying and exploring the boundaries created by illness, and it fluctuates as life and illness itself presents new challenges ([15], page 265).

Despite increasing emphasis on the need for structured disease-specific patient education programmes in severe chronic illness [19], only a few studies on patient education in the area of psoriasis are available [20–24]. The research in this area has tended to focus on the importance of the physician-patient relationship to improve the patients' perceived control of the disease [20, 22] as well as information on psoriasis and treatment as a way to improve adherence to treatment, skin care [22, 24] and psychological relief [23]. One disease management programme focused on knowledge of the disease, patient skills training and psychological support [24], documenting improvements on disease severity, adherence to treatment and quality of life. Although the inclusion of psychosocial aspects is recommended in structured education programmes [5, 25, 26], one review [27] reported a lack of evidence of the effect of psychosocial interventions for individuals with visible differences including psoriasis.

With one exception, the above-mentioned studies reflect the health professionals' perspective of what is important in educations programmes. Less attention has been paid to the patients' perspective and substantial educational needs in daily life. Linder et al. [22] identified hope of curability, perception of control and understanding from the physicians as valuable elements for the patients. Generally, the studies provide no clear recommendations related to content, pedagogical considerations, patient involvement, or outcome measures.

In nursing science, Thorne et al. have shown that patients depict a specific “world of disease” for each chronic disease—a cultural knowledge of the implications of the illness and the particular care for that world [19, 28]. This cultural knowledge greatly influences how information about the disease and health is received by patients and consequently should be taken into consideration when developing patient education. A similar viewpoint is reflected in a recent Danish Health Technology Assessment (HTA) about patient education [19]. The HTA recommends that the patients' experience of the specific nature, characteristics, and course of disease should be involved when developing new interventions. This means that a new intervention should focus on the needs of the patient, thereby ensuring patient-oriented criteria for success as an important factor determining the content of the programmes.

To our knowledge, no studies have yet investigated the needs assessments related to patients with psoriasis. According to Bartholomew et al. [29], the initial process of needs assessment may include a systematic study of the scientific literature reporting on the discrepancy between what is and what should be, experienced by patients living with a problem like psoriasis. This discrepancy should be studied in relation to quality of life, health status, and factors that influence health and health risk. These factors include lifestyle, health behaviour and social behaviour, personal factors like self-care, coping and adaption as well as family as part of the environmental factors.

Therefore, we designed an integrative review to gain insight into and achieve a comprehensive understanding of people living with psoriasis and their needs for patient education based on their perspectives. The review aimed to answer the question: which factors related to the disease and the course of disease may, according to the existing literature, impact patients' needs of structured patient education to support self-management of psoriasis in daily life?

## 2. Methods and Materials

An integrative review of the literature was conducted, applying the strategy for this method described by Whittemore and Knafl [30, 31]. The method was chosen because qualitative and quantitative studies can be combined within different disciplines. Consistent with this approach, the review included (1) problem identification (stated previously), (2) literature search, (3) data evaluation, (4) data analysis and synthesis, and (5) presentation.

### 2.1. Literature Search

A search strategy was designed in cooperation with a research librarian. Searches were conducted in February 2010 and repeated in January 2011. The following databases: Bibliotek.dk (Danish), PubMed, Embase, CINAHL, and SveMed+ were searched for papers in English or in a Scandinavian language published between 2000 and 2010/2011. A complementary search in PsycINFO was conducted in September 2012. Variants of the following keywords were searched separately and in combination, adapted to the thesaurus of the database concerned: *psoriasis AND quality of life OR health status OR lifestyle OR health behaviour OR social behaviour OR family OR self-care OR coping OR adaptation AND research OR nursing research OR interview OR focus group OR qualitative research OR questionnaires OR survey*. Further references were searched for using reference lists in retrieved papers, and relevant nursing journals (Dermatology Nursing, Scandinavian Journal of Caring Science, Patient Education and Counselling, Chronic Illness) were checked to identify other relevant studies.

#### 2.1.1. Selection Criteria

In addition to the main inclusion criteria mentioned above, further criteria were determined as they captured the focus of the research question. The papers should report on either the patients' perspective on the impact of psoriasis on their lives or their support needs to be able to manage the illness in everyday life. The papers were excluded if they (1) reported on medical treatment using quality of life measures as well as the development of measuring instruments, unless the patients' perspective of the impact of disease or self-management was clearly stated, (2) included several groups of patients unless the results for the psoriasis group were reported separately, or (3) reported separately on psoriasis arthritis, as arthritis has a different impact on function, fatigue, and pain than is the case with skin symptoms and therefore gives rise to different needs related to joint protection.

#### 2.1.2. Data Evaluation

The titles and abstracts of all studies identified by the search strategies were assessed for their relevance to data extraction. Those that immediately met the inclusion criteria were selected for more detailed examination. The full-text versions of these studies were then obtained for close reading. The full-text reading caused the exclusion of papers that were not considered relevant to this study, for example, papers reporting on telephone consultancy methods or different models of illness and their suitability in medical practice, socioeconomic impact of disease, association between psychological variables and outpatient services. Due to the substantial diversity of the methodological approaches, we developed two quality criteria instruments according to recommendations by Polit and Beck [32] and Forsberg and Wengström [33] (see Table 1).

**Figure 1:**
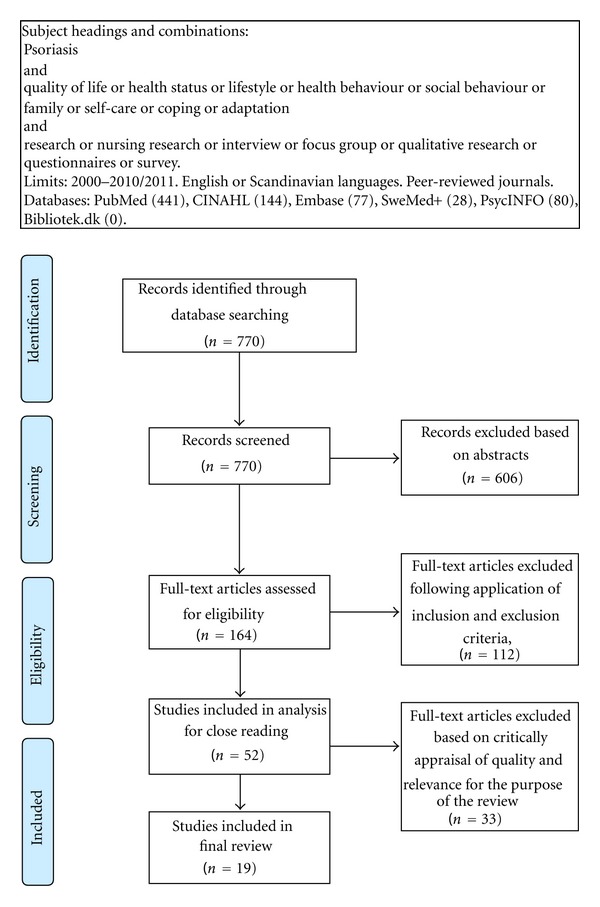
Flow diagram of the search process, adapted from Moher et al. [34].

The instruments were developed for the qualitative and quantitative studies, respectively, enabling us to assess methods of research design, sampling, and conclusions. To prevent unnecessary complication of the analysis process, the quality of each included study was assessed as good or less good. Papers were included for final review if they generally met these criteria. A total of 770 papers were identified by key search terms, but most of these failed to meet all inclusion criteria (see Figure 1).

#### 2.1.3. Data Analysis

According to the research strategy, each paper was carefully examined for data reduction, data display, data comparison, conclusion drawing, and verification [30]. The first phase of the data reduction classified the papers in terms of author, year of publication, country, research design, and disciplinary perspective. The disciplinary perspective was determined by the professional title of the first author and the name of his/her institution, and verified by web search if needed.

The second phase of the data reduction was performed according to aim, informants or population, data and measures, findings and results, and, finally, the authors' conclusion and discussion. For each paper, data relevant for the purpose of this study were extracted and organised in comparable frameworks. Thus, each primary source was reduced to a single page with similar data. Because of the fragmentation between the studies we found no heterogeneity of the above-mentioned variables, which is why an overall comparison of the studies would not make sense for the purpose of this study. We then proceeded inductively and developed a coding scheme where the objective of the coding is in the realm of themes and ideas [35]. In this approach, the objective of the coding is to develop a broad-based code, for example, physical symptoms from the skin in order to create collection of accounts, for example, pain, itching, scaling, discomfort, or not well at all, from which we could begin considering whether the phenomenon refers to illness, the trajectory, the treatment, the life phase, and so forth. The coding included 13 themes of significance to patient needs (see Table 3). The themes were divided into three separate clusters, and each paper was examined with the purpose of verification of coherence with the clusters (see Table 3.) To establish a clear connection between the clusters, each of the three clusters was further explored using the original data material to identify differences, similarities, and internal relationships. This in-depth analysis was synthesised into an interlevel model ([36] page 146) with the intention to present the complex interaction of high impact factors at different (psychological, social, and biological) levels related to the disease and course of the disease that should be addressed in future structured patient education programmes in order to support the patient' self-management in daily life.

## 3. Findings

Nineteen papers were included for final review, representing different countries: Norway, UK, Poland, The Netherlands, USA, Sweden, South Africa, Australia, and three disciplines: medicine, psychology, and nursing. Seven papers represented qualitative studies, while 12 papers represented quantitative studies. All selected papers address, in different ways, the patients' experience of discrepancy between how life is lived with psoriasis and how life should be lived. The purpose of the studies fall into two main groups: (1) to investigate the perspective of patients and/or families/partners on the impact or the burden of disease, and (2) to investigate the association between several different factors that influence the patients' ability to manage the disease in relation to function, well-being, and quality of life.

Different theories were applied in the studies, including Lazarus' & Folkmann's coping theory, Leventhal's common-sense model as well as various theories on pain, self-concept, stigma, illness acceptance, and body phenomenology. A wide selection of measuring instruments were used, with Psoriasis Area and Severity Index (PASI) and Psoriasis Disability Index (PDI) being the most recurrent.

Three main clusters of high-impact factors were (1) disease-specific factors constituted by visibility of disease, onset time and the fluctuating nature of disease; (2) self-experienced burden of disease, where the physiological, psychological, and social self-experienced burden of disease were the most frequent themes in most studies; (3) personal factors with themes like illness perception, coping strategy, gender, age, family, and partners. Table 3 shows the distribution of the selected studies on the three clusters (numbers indicate reference number in this paper). Several studies appear in more than one cluster.

### 3.1. Disease-Specific Factors

#### 3.1.1. Visibility of the Disease

Visibility is distinctive for the nature of the disease and significantly influences how patients manage their disease. In a trans-European study, 48% of the patients reported that their quality of life was affected in relation to physical appearance [48]. For both men and women, the greatest difficulty of living with psoriasis was the sense of being marked by the disease [38, 49]. Such experiences are described as a sense that other people tend to scrutinize and judge them, their character and inner world according to the appearance of their skin [53], or more correctly their own perception of this appearance [38]. In particular, the patients are worried that they may be perceived as having low hygienic standards, or that the disease is contagious [53]. This perception is especially distinct in patients who have experienced teasing and bullying during childhood [49, 53]. The sense of being marked by the visibility of their disease was like an ever-present shadow for the majority of the patients, especially in out-groups and public places [49]. Visibility emerges as a general theme throughout the material to such an extent that it appears in all three clusters.

#### 3.1.2. The Fluctuating Nature of the Disease

The disease is characterized by its fluctuating nature and thereby a fluctuating course of disease/trajectory. Patients experience their disease as a physical, tumultuous journey while the disease expands and one flare-up is followed by another [51], or as an eternal cycle where the patient at one time can function normally and the next moment finds himself almost isolated from the world. A symptom-free period can be experienced like being released from prison [38]. This fluctuating course of disease means that, when an accumulated loss of earlier self-perception cannot be restored and replaced by a new image, the patients may come to experience reduced self-esteem and self-confidence [38, 51].

#### 3.1.3. The Significance of Onset Time

A number of studies suggest that the patient's age at onset time for the disease is perhaps the one factor with the greatest influence on the course of the disease [49, 53]. This is probably linked up with the fact that the process of being marked begins very early. The sense of being young and single and suffering from an “ugly” disease like psoriasis is described as devastating. When they looked back, the patients related that, in their youth, they looked on their body with disgust and assumed that nobody would ever wish to be their partner [51].

One study points out that, in addition, the time immediately following onset is a significant factor for the course of disease [51]. This period can be filled with the fear of having to live with the disease and for the ensuing consequences. The understanding of the chronic nature of the disease and that one cannot be cured, either by one's own effort or with the help of others, is described as very depressing. These worries were felt most strongly by patients below the age of 30 and were expressed as thoughts of having no partner, job or close friend to rely on [49].

The studies show that fundamental conditions such as the visibility and fluctuating nature of the disease are of crucial importance to patients, not least if the onset occurs during adolescence.

### 3.2. Self-Experienced Burden of Disease

Patients with psoriasis suffer considerably from the impact of the disease on daily life as regards general health-related quality of life, disease-specific quality of life as well as quality of life as lived experience [37, 47–49]. In a Norwegian study, the patients reported a reduced general health-related quality of life within all of eight conceptual domains including: self-reported general health, physical functioning, bodily pain, mental health, physical role limitations, emotional role limitations, vitality, and social functioning [37]. Among European patient association members, 70 per cent of the respondents stated that the disease generally impacted their lives [48]. One study shows that patients, even after having lived many years with the disease, experience that psoriasis impacts their daily lives [49]. The studies distinctly indicate that the clinical severity of the disease has no causal relation to how patients experience the burden of their disease and the impact on their quality of life. It is the self-experienced severity of symptoms and outbreaks that constitute a key factor in relation to the course of disease and are significant for how well patients manage to live with their disease [37, 48].

#### 3.2.1. Physical Burden of Disease: Itch, Pain, and Discomfort

Research in this field has mainly been focusing on patient perspective on the psychosocial burden of psoriasis rather than on how physical symptoms affect daily life. However, a frequently cited study on patients suffering from severe disease indicates that the disease is associated with an extensive experience of bodily suffering, physically described as pain and reduced mobility [38]. Recent studies indicate that the physical burden represented by itch [52, 54] as well as skin pain and discomfort [55] is likely to be more serious than has previously been assumed in cases of severe and mild disease.

In particular, several studies report itch to be one of the most unpleasant and irksome symptoms related to psoriasis. Itch is described as unbearable, worrisome, bothersome, and annoying, and the sensory dimension is described as burning, stinging, and crawling [54]. The sensory experience may be severe and may affect power of concentration, daily activity, physical activity, sexual activity, and sleep [52, 54]. One of the studies accentuate the need for the development of instruments that are capable of measuring itch in clinical practice, in order to better help the patient group manage this severe problem initially by acknowledging the problem itself [54].

The treatment as such is reported as bothersome and ineffective [48] and is experienced as unpleasant, painful, and draining the patients of time and energy. This applies to daily home treatment, hospital or clinic treatment as well as side effects such as itch, pain, smell, and cosmetic inconveniences. The patients describe a balance between treatment or no treatment of the skin and a feeling of being caught within the paradox of being damned if they treat the psoriasis, and being damned if they do not [38, 51].

#### 3.2.2. Psychological Burden of Disease: Change of Self-Concept

A number of studies state that patients with psoriasis suffer from an increased level of anxiety and depression [37, 40, 44, 48]. The findings of these cross-sectional studies leave no doubt that the disease results in psychological implications. However, these findings are based on measurements developed chiefly for psychiatric diagnosing, and the study designs are not suited for unearthing causal connections and slight nuances in patient perception. In an Australian study, the patients' descriptions of their experiences of depression and anxiety did not reflect the symptoms as signs of clearly defined psychiatric diagnoses but rather as causally connected with experience of shyness, shamefulness, or social limitation related to the localization and visibility of the disease [53].

Likewise, studies investigating the lived experiences of the patients report that causal connections are complicated when trying to understand the psychological implications of the disease. The bodily changes caused by the disease and the degree of visibility may result in a changed self-concept. Patients experience that psoriasis has no boundaries in its assault on the physical body and perceive the disease as an enemy attacking the physical self [51]. They describe their bodies as being offensive, unclean, infectious, disgusting, leprous, ugly, unattractive, strange, or different [38]. The attack on the body is experienced as ungraceful, embarrassing, and humiliating, which may result in a feeling of helplessness and powerlessness, especially when not knowing the causes and implications of the disease or their possibilities for getting help or solace. It is concluded that psoriasis may be radically life-changing for the individual's relation to him-/herself and others, thereby resulting in deconstruction and self-fragmentation [51].

This changed self-image has a considerable variety of feelings attached to it. Such feelings may be despair, melancholy, aggression and vulnerability [51], depression and anxiety [41, 45, 53] as well as shamefulness. The highest emotional burden was found in women, but also men were clearly affected by worries about visibility and experience of limitations in relation to social and sexual activities [49, 53].

#### 3.2.3. Social Burden of Disease: Change of Self-Concept Results in Social Vulnerability

Several studies report that the disease considerably influences the social functioning of the patient group [37, 47]. This includes the impact on aspects related to holidays, travelling and making new friends [45], leisure activities such as sports and gymnastics, jobs and school attendance [48] as well as beauty care and cultural events [53].

Self-concept in interaction with reactions in the patient's surroundings and the patient's interpretation of such reactions may lead to social vulnerability. The patients transform their own thoughts and feelings about the look of their skin to other people and suffer from what they think other people think of them [38]. Most patients have the perception that their body no longer supports daily social activities.

Patients describe the fear of being rejected in social contexts as a considerable factor. The fear of being rejected is expressed as a strong wish to hide the disease, to make it nonvisible. Patients talk about respecting the rules of psoriasis in terms of diet and clothing [51]. The patients' struggle to conceal the disease is very time and energy consuming and may often result in considerable restrictions as regards lifestyle and daily activities. In particular, scaling is a nuisance to the patients in daily life, entailing the frequent changing of clothes, increased laundering and, not least, daily cleaning to remove scales from beds, floors, and chairs [38, 49, 53].

A number of studies point out that the disease affects the entire family of the patient. The patients report that, during periods of disease activity, they experience themselves as a burden to their closest family [38]. A recent English study reports that many aspects of patient or family life may be affected. This primarily includes added house work in relation to skin treatment and removal of scales. Other aspects in this context were worry, social limitation due to embarrassment or the patient's need for help, leisure and holiday limitations as well as stressful relationships to the patient and other people. Finally, the informants pointed out impact on their sexual life [50].

To summarize, the analysis shows that the degree of self-experienced severity and the assessment of the importance of the symptoms are crucial to how patients experience the burden of disease. The analysis shows that the disease may deeply influence daily life for the patients as well as their families/partners. Body perception and self-concept is changed in a way that increases the social vulnerability of the patient group. The fear of being rejected stands out as a considerable factor for planning and activities in daily life. The analysis also shows that in addition to visibility, itch, and skin pain are important in relation to how restrictions are perceived in everyday life.

### 3.3. Personal Factors

The included studies tend to point in two different directions: (1) one direction stipulating that knowledge of the patients' perception and interpretation of the disease is an important starting point, and (2) another direction emphasizing the knowledge of specific coping strategies of the patient group as a fundamental precondition for being able to help and support patients with psoriasis.

#### 3.3.1. Illness Perception

Several studies state that illness perception is more important for how patients perceive functioning limitations than the extent and severity of the illness *per se*, and also state the existence of an association between illness perception and self-experienced psychological problems [40, 43–45]. Illness perception is constituted by the patient's perception of potential causes for the disease, experienced consequences of the disease, perception of or belief in recovery and control of the disease as well as disease identity.

One study showed that medical treatment of psoriasis affects physical functioning and perception of disease-specific stress but does not affect the patients' worry, perception of the disease, or strategies for managing the disease. Even in cases of considerable improvement of the clinical severity of the disease, the patients' perception of psychological problems and coping strategies, for example, concealing, did not change [44]. This may be connected to the fact that worry and anxiety have been related to cognitive aspects such as illness perception rather than behavioural aspects such as coping strategies [44, 45].

Some studies show that more than 50 per cent of the respondents believe that personal causal connections such as stress may result in psoriasis and subsequent outbreaks [42, 43]. Patients who believe that their disease is emotionally determined experience a considerably larger degree of morbid worry than patients who believe that their disease is determined by physical causes [44]. Stress as an assumed cause is associated with a low level of psychological well-being [43] and reduced knowledge of self-care actions [42]. An English study reported that inequality between the way patients and partners think about the disease may affect the psychological well-being of both parties as well as the clinical result [45].

#### 3.3.2. Coping Strategies

Studies within nursing [46], psychology [39], and medicine [49] have investigated how patients cope with their disease in daily life. The studies are based on the coping theory developed by Lazarus and Folkmann as it offers a perspective that acknowledges the fact that patients develop coping strategies that interact with their environment. The studies presuppose that it is important to be able to understand the line of action taken by the patient in order to be able to provide support and care for the patient.

One study investigating general ways of coping with chronic medical conditions shows that patients with psoriasis did not apply coping strategies nearly as frequently as patients with chronic fatigue, atrial fibrillation, spinal cord injury, cancer, or myocardial infarction, especially with regard to social support [39]. This is interpreted as an indication that many of the problems experienced by the patients surpass the coping resources of the patient as well as his/her family and social network. Another study shows that having a family, a job or a close friend, being useful or feeling well were factors that facilitated coping and were able to make patients forget that they were suffering from psoriasis [49].

Results from a Norwegian study specifically investigating how individuals cope with severe exacerbation indicate that disease duration, age, and gender are important for the choice of strategy. Women and adolescents use emotional strategies more often than men and elderly people. Disease duration is important for how patients choose to act. The longer a person has lived with the disease, the more often a believe-in-yourself strategy is applied. The opposite is seen in relation to supporting strategies: the shorter the disease duration, the more often a supporting strategy is applied [46]. This indicates that individuals who had lived longer with psoriasis developed personalized knowledge about disease management that made them more independent of support to manage problems from other people. However, the findings from another study indicate that patients, irrespective of gender, choose the strategy that fits into their daily lives. In order to adjust themselves to the disease, the patients transformed the treatment and concealment strategies to everyday routines such as teeth brushing [49].

To summarize, the studies contribute to understanding that cognitive factors, such as illness perception and adequate choice of strategy to manage disease in daily life, have importance for the course of disease. However, patients with psoriasis do not apply coping strategies nearly as frequently as patients with other chronic diseases. The studies indicate that individuals with psoriasis tend to explain their disease based on personal causal connections, with a resulting negative impact on functioning and life quality. The studies also indicate that adolescents are in most need of support to manage their disease, while elderly patients and patients who have suffered from the disease for a long time develop a believe-in-yourself strategy and transform treatment and concealment strategies to everyday routines.

### 3.4. Synthesis

**Figure 2:**
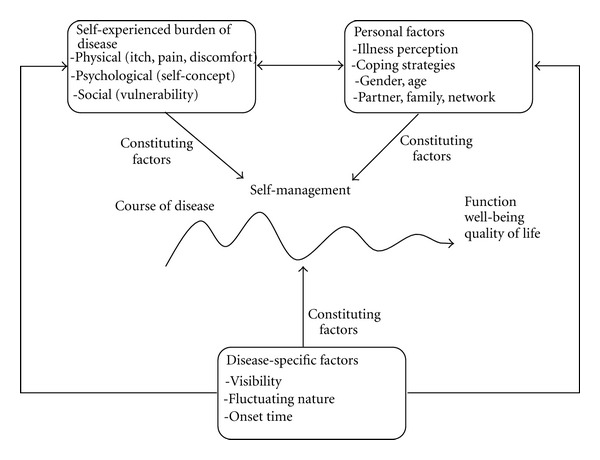
High-impact factors for patient self-management of psoriasis in daily life.

The analysis indicated a number of high-impact factors that constitute the course of disease and consequently its influence on quality of life, function, and well-being for patients with psoriasis. A synthesis of the findings is shown in the interlevel model in Figure 2.

Figure 2 illustrates that course of disease is constituted through an interaction between various factors, grouped as disease-specific factors, self-experienced burden of disease, and personal (and environmental) factors. For each group, the model lists a selection of factors which, in their own right and in a complex interaction with other factors, influence on how well each patient manages his/her disease in everyday life and is able to achieve better functioning, well-being, and quality of life.

The data comparison demonstrated that visibility is a factor of radical importance to self-perception, social vulnerability, and daily activities in general. It is not the clinical extent of psoriasis, but rather the visibility-associated severity experienced by the patient that determines how severely the burden of disease is felt and which strategies the patient chooses for managing the disease. Furthermore, our analysis suggests a connection between the patient's perception of the disease and how the burden of disease is felt, and that this will influence how the patient chooses to act. The burden of disease is felt more profoundly if the patient's comprehension of what causes the disease is founded in a personal belief of cause and effect. The analysis also suggests that personal relationships with family and partner are significant with regard to social vulnerability and experienced burden of disease, and that quality of life for the patient and his/her partner is influenced by differences in perception of the disease.

The analysis of disease-specific factors suggests that the age at onset time and the time immediately following onset are significant determinants of successful self-management. Early onset influences the burden of disease and, seemingly, personal factors such as illness and body perception, while onset during adulthood does not result in the same degree of emotional strain. The fluctuating nature of the disease is a particular disease-specific factor. The self-experienced severity of the disease and the frequency of fluctuations of the disease seem to influence how well the patients will recover in each period between exacerbations, as well as the patient's self-perception and psychological problems.

## 4. Discussion and Conclusion

The findings from this integrative review of factors that may impact patients' needs of structured patient education reflect complexity, both in terms of the impact of the disease on the patients' health and quality of life and the specific cultural knowledge of this patient group. The model does not provide any answers as to how learning processes should be organised but contributes to the development of understanding and presentation of health problems in general in this patient group. Not least patients for whom it represents an excess burden, which in fact is the product of the first step in a needs assessment [29]. For this patient group in general, the most important lesson to be learned from this review is that patient needs must be understood in a complex interaction between self-experienced burden of disease and its visibility, personal conditions such as illness perception, and the patient's age at onset time. These factors appear to be of importance when considering the second phase of a needs assessment, where the connection between the needs and the programme outcomes is established [29], and within nursing, it is necessary to discuss the most appropriate methods and strategies to address the factors.

The experiences of being marked by the visibility of the disease was a finding we had anticipated, given that one in four patients experience significant psychological distress, and a perception of stigmatisation has been reported to be one of the most significant factors related to disability and quality of life [56]. However, the research on stigmatisation in this area has tended to focus on associations between various personal variables such as depression, age, gender and age of onset and to a lesser degree on the social processes reflected in our data. The findings indicate that an overly narrow focus on psychological implications may be problematic because it will always be attached to the individual. It has been discussed that if stigma is viewed as a personal property instead of a label attached by other people, this may have a decisive effect on where the responsibility is placed and, consequently, which interventions are applied [57]. This schism between, on the one hand, health professionals focusing on easing the patients' emotional reactions to their disease and, on the other hand, patients focusing on letting their experiences be expressed as routine actions with the purpose of hiding their disease seems to surface the tension that exists between what the health care system is offering and what patients think they need. Patient education interventions have often been organised in accordance with the needs of the clinician and the system taking priority in the delivery of patient care. In models like this, the professional is at the centre of the system, and the patient is expected to comply with the instructions given by the health care professional. If our findings are seen in a self-management perspective, they indicate that visibility can, with advantage, be understood in relation to how patients act on it as part of their struggle to create order and control in daily life.

In agreement with previous research within other chronic conditions [58] patients' causal beliefs of the illness and its consequences appear to be of greater importance for the patient group's health-related quality of life than the factual clinical severity of the illness. Worry and anxiety related to illness perception may limit the patients' choice of alternative points of view on themselves, their world, and their future. Nevertheless, illness perception must be emphasised as an important factor in relation to psoriasis patients, as in two of the studies 60 and 54 per cent of the patients, respectively, stated person-specific factors like stress as being the cause of their disease [42, 43, 59]. This is confirmed by a review [56] showing that 37–88 per cent of the included patients stated stress as a disease cause. Recent stress research confirms that a majority of patients believe this to be true, but clearly states that stress cannot be established as a triggering factor in relation to psoriasis [56].

This discrepancy could be due to the fact that the disease has not, until recently, been recognised as an autoimmune-mediated disease. For many years, it was a prevailing hypothesis that patients with psoriasis had a certain style of personality [40, 56]. From this outset, health professionals have sought explanations for the disease in the patients' personal factors and stressful life events, and it has been suggested that health professionals may have contributed to creating this understanding [43]. For psoriasis patients, this understanding may have resulted in being particularly vulnerable to topics such as psychological condition and lifestyle, given a basic perception that they themselves are the reason for the onset and flare-ups of their disease. It is already known that patients with a disease of unknown causality are vulnerable to communication related to their psychological condition [28]. Historically, patients may have adapted the perception that no doubt has unconsciously been signalled by the health professions. Thus, the patients' illness perception is an important factor that should be taken into account when appraising the patient group's needs for patient education. Here lies a commitment to communicate the latest findings on disease causality if such knowledge prevents psychological difficulties.

The Danish Center for Health Technology Assessment [19] found that people with chronic diseases need disease-specific knowledge and skills. Still, what is supposed to happen within the communication to steer the patients' course of disease is not at all well-known, and the HTA does not provide any comprehensive overviews of these needs. It is pointed out that the focus should be on the patients instead of the health care professionals' assumptions about the needs and the preferences of the patients. This means that the nursing discipline must critically discuss the customary way of defining clinical outcomes and the outcomes that are important for patients to live well with their disease. Thorne warns against the evaluation of interventions in terms of available indicators of treatment compliance and lifestyle control [60] and challenges nursing to adopt the perspective of expert patients; that there is no single way to live well with a chronic condition; that the learning process is complex and stepwise; and that the role of nursing science must be contextualised within an understanding of the living that is taking place [61].

There is emerging evidence that interventions that specifically aim to increase the patients' level of self-efficacy are more likely to produce positive outcomes in terms of behaviour change and health outcome. De Silva [62] for example, has reported that people with chronic conditions benefit from different outcomes like improvement of knowledge, technical skills, self-efficacy, and behaviour change. It may however be questioned whether these existing outcome measures really capture the benefits that are most important to the participants in the programmes. If this is not the case, many important factors can get left out when the impact of a programme is evaluated.

Our findings indicate that the outcomes must reflect the factors that may be important for the individual patient, both in spontaneous patient education and in planning for a more structured process. Outcomes must reflect how far the patient is in his or her course of the disease, how the burden of disease is experienced, how the body and illness is experienced, and the best ways for the patient to manage the disease. It seems that varied approaches are needed and that researchers in nursing science must build relationships with the patients and engage them in the research to map the outcomes which really benefit their lives.

According to the theoretical concept behind needs assessment and health promotion intervention, “nothing is as useful as good theory” ([63] page 8). This means that when developing patient education programmes, the choice of a (multi-)theoretical framework contributes to a coherent intervention development addressing the content, the pedagogical methods, the organisational structures, the competency development of the health care professionals, and the development of outcome measures. However, based on the findings of this review it could be important to question whether these theoretically defined outcomes really meet actual patient needs.

Kennedy and Phillips argue for broader and more patient-centered measures to capture the social impact of patient education programmes [64]. They have shown that participating in a well-defined and evidence-based programmed as the Expert Patient Program [65] improved the participants' confidence, knowledge, and skills as a part of the programme curriculum, for example, improved diet, meeting new people, ability to control emotions, increased self-awareness, and increased self-worth. But these outcomes were not those valued most by the participants [64]. In focus group discussions with participants, the ancillary (not health-related) impact of the programme was mapped, and a general “theory of change” for this particular patient group was developed and tested. It seems that an increase in confidence leads to further outcomes such as decreased anxiety, better sleep, the ability to try new things, and increased motivation. Thus this method permits the mapping of variety of outcomes experienced by different participants, for example, improved relationships with family and friends, participating in volunteering initiatives, further education, or job-related outcomes.

Our findings indicate that when evaluating the patient group's general needs for patient education, the health care professionals must pay more attention to the various life phases where psoriasis sets in. This issue has not yet been focused on in a scientific context, and onset time is not an articulated theme in the included studies. The findings can be seen in the light of recent knowledge that the disease sets in either at an early age or relatively late in life. More than half of the patients experience onset before they reach 20, in most cases with the first symptoms appearing before the age of 16. For the majority of the rest of the patient group, onset does not occur until the age of 50 to 60. The group with early onset is characterised by a higher severity of the disease, while the group with late onset is characterised by lower illness severity [2, 66]. Our data material is characterised by no inclusion of very young people in any of the studies. The average age for the included patients was between 40 and 52 years. Several studies included middle-aged patients with early onset, a fact that stands very clear in their memory. In the studies, the patients recounted in retrospect their fear of never being able to get a partner, a job, or a close friend, and that nobody would ever come to like them [49, 67]. These stories illustrate that onset during adolescence was a great strain on them, and described as devastating. Our review indicates that patients with onset during adolescence had by far more difficulties with managing their daily life than patients with adult onset, especially with regard to psychological difficulties related to body and self-perception. One study found that psychological difficulties were particularly distinct in individuals who had experienced teasing or bullying as children or adolescents [53]. Research in young people with psoriasis is indeed limited, but a theoretical study indicates onset time to be a decisive factor for development of psychological difficulties [68]. In accordance with our findings, this study describes the onset of the disease in late childhood being experienced as a stranger and therefore a frightening experience. Patients in this developmental phase have a strong wish to understand what is happening to them. The study also points out that patients who do not get sufficient support to socialise with their contemporaries often tend to develop emotional problems and academic difficulties as adults. Findings from an online focus group survey [69] including young people between 11 and 18 showed that the young people struggle to make the disease fit into their lives. It is a struggle between “it” and “me”, and the struggle is about controlling “it” and minimising its influence on their appearance and social functioning. Through the research process when articulating their experiences, the young people became aware that they were not alone in their struggle and were spontaneously practising *peer support* to help each other. The study argues that the resources achieved during adolescence are significant for the ensuing adult life phase. It seems likely that the potentially negative long-term effect of psoriasis may be reduced by participating in network groups during adolescence.

It appears that onset time is a high-impact factor that must be integrated in the planning of structured patient education programmes, taking into account that onset time is related to two very different age groups with different life phase challenges. In particular, it appears that we must increase our focus on the problems and the vulnerability related to having a visible disease such as psoriasis during childhood and adolescence and acknowledge the need for a preventive intervention at an early stage.

Overall, our findings indicate that further research is needed on the needs of patient education programmes. The research would benefit from the involvement of especially the young people in particular to explore their specific needs including specific and various outcomes relevant to their everyday life. When participants are involved in the designing process of patient education, they prioritise the educational content very differently from their teachers. Booker et al. [70] argue for development of educational interventions in cooperation with the patients using focus group discussions to articulate problems and outcomes. This is important both in relation to children and parents, as children need to understand what is happening to them, and in relation to adolescents during the critical period of developing their identity when they not only need family support but, to a greater extent, compare themselves with their contemporaries.

### 4.1. Study Limitations

This review has some limitations due to the complex method. On the one hand, the preliminary search of this review indicated limited research on nursing and nursing intervention within this patient group. On the other hand, the search revealed a widespread research reporting on the impact on quality of life, function, and well-being. The establishment of the theoretical scaffolding around “needs” ensured well-defined key words and improved the sensitivity of the search. This resulted in an extensive data material that enriched the understanding of patients' needs but challenged the selections and analytical process. Ideally, all relevant literature should be included in the review, and more studies might have been included to contribute to data saturation. However, a high degree of retrieval throughout the search process indicates that the review constitutes a representative picture of the available scientific knowledge on the phenomenon [32].

Given that the review is based on original studies from different disciplines and methodologies, there might be some limitations with regard to the data extraction. Although we have sought to be compliant with the research tradition, methods, concepts, and theories of the various disciplines, this complexity may have constituted a risk of misinterpreting the quality and the findings of some studies. However, we have put the interpretation of the existing knowledge into the context of current health care and into a theoretical understanding of the patients' perspective on self-management. This may contribute to the cumulative knowledge in nursing science and practice [71].

The review may also be confined due to the complexity of the data evaluation. Several of the primary sources contained weaknesses with regard to the selected quality criteria, but overall the quality was high. The studies that were valuated less good were to a lesser extent used in the analysis, for example, when the statement of the problem was not quite clear or the questionnaire was only partially validated.

It can be discussed whether our findings concerning the young people are transferable to present time, considering the relatively high average age and illness duration of the participants as the participants' experiences in most cases were formed in contexts more than a generation ago. It is questionable whether psychosocial difficulties experienced by the participants during adolescence would be similar today. We have found the difficulties to be transferable to present time, as body experience and self-representation during adolescence is more important than ever in Western countries.

The integrative review method is not different from other systematic review methods with regard to the fact that bias and errors may occur at any stage of the review process, and that attempting to eliminate all biases would be naïve. What is characteristic for this particular method is its ability to comprehend knowledge about a phenomenon in various disciplines and methodologies and the use of inductive analysis methods. This means that its foundation is not collection and comparison of evidence but an interpretation of existing knowledge. The aim of this analytic form extends beyond taking things apart and putting them back together again [35]. We present an interpretation that has the potential to contribute to present varied perspectives of the patients' needs and is thereby important to both nursing practice and nursing science. We have tried to ensure the validity and transferability of the interpretation by presenting the rather comprehensive (Table 2) that shows the data extraction of key data reported as thoroughly as possible, using the original language and expressions of the studies reviewed.

### 4.2. Conclusion

The aim of this integrative review has been to identify and describe patient needs for education to support self-management in daily life with psoriasis. We have identified a range of high-impact factors associated with the disease and its course that substantially influence the quality of life, functioning, and well-being of the patient group. These factors constitute, in mutual complex interaction, the course of disease and are significant with regard to how well the patient is able to manage his/her disease in everyday life. The factors are grouped into three clusters consisting of disease-specific factors, personal factors, and self-experienced burden of disease. We conclude that some of these factors may be particularly important to take into account when developing education programmes for this specific patient group. Among these factors are the patients' illness perception, how the visibility of the disease influences the psychological and social burden of the disease, and, not least, the particular importance of onset time, especially for the young patient group.

The study offers a profound understanding of what may be important to patients participating in self-management education, and the interlevel model offers a contribution to nursing practice to encourage nurses to involve patients in the development of new interventions.

## Figures and Tables

**Table 1:** Criteria for assessment of identified papers.

Qualitative papers	Quantitative papers
Problem stated unambiguously?	Statement of the problem?
Ethical problems reflected?	Identified aim, hypothesis or research question?
Identified aims congruent with methodology and methods for data collection and analysis?	Sufficient description of the population and transferability into Danish context?
Sufficient details of the informants and settings?	Adequate description of measures?
Method for data collection described?	Adequate description of data collection?
Sufficient detail of the process of analysis provided to ensure accordance with selected method?	Sufficient description of data analysis?
Results presented systematically?	Findings adequately summarised and answering research question?
Conclusions reflect results from the study and relevance for practicing nursing in a Danish context?	Generalisability of the findings for Danish nursing practice and discipline?

**Table 2:** Key data, selected papers.

No.	Author, Year, Country, Discipline, Design	Aim	Informants, Population	Data, Measures	Findings, Results	Author conclusion and discussion
[37]	Wahl et al., 2000. Norway. Nursing. Cross-sectional.	To assess health-related quality of life among patients with psoriasis and to compare these estimates with population norms.	283 patients treated at 3 dermatology departments in the eastern part of Norway. Gender: 57% M, 43% F. Mean age: 47 years. Control subjects 3.500 of the general. Norwegian population, mean age: 45 years.	Short Form 36	After adjustments had been made according to age, gender, educational level and marital status, it was seen that psoriasis patients reported poorer health-related quality of life in all 8 conceptual domains compared to norms from the general Norwegian population. The largest difference was found on the role limitation scale—emotional scale.	The burden of psoriasis is significant at the emotional life domain. This domain deals with problems in relation to work or other regular daily activities as a result of any emotional problems, such as feeling depressed or anxious. The patients' own assessments of symptoms specifically related to psoriasis are related to their evaluation of health-related quality of life.

[38]	Wahl et al., 2002. Norway. Nursing. Qualitative.	To present results from a qualitative study that focuses on patients with psoriasis, their experience of living with the disease, and its psychological and social impact.	22 hospitalized patients with severe disease, that is, more than 30% of the body affected. Gender: 10 M, 12 F. Age: 20–80 years (majority 40–60 years). Diagnosed with psoriasis for more than 3 years.	Transcribed interviews.	Bodily suffering emerged as the core category, with the following subcategories: (i) The visible body (ii) Social vulnerability (iii) Staying on an even keel (iv) An all-consuming disease Many different aspects and nuances are related to the patients' body experiences, for example, concern that their body seemed offensive, unclean, infectious, disgusting, leprous, ugly unattractive, strange or different. The wish to keep the disease secret and to camouflage it is a very prominent aspect of living with psoriasis. Feelings of despair, vulnerability and hopelessness are related to living with psoriasis. Psoriasis can control every aspect of the patients' lives.	Patients' experience of living with psoriasis includes bodily suffering, changed self-image, the perception of being stigmatised, and social isolation. For some people, the body can no longer support the positive social function. Psoriasis is described as an enemy that saps the patients' spark and energy during bad periods.

[39]	Fortune et al., 2002a. United Kingdom. Psychology. Cross-sectional.	To examine strategies for coping in patients with psoriasis and investigate whether they differ compared to normal controls and patients with other major medical diseases.	250 patients attending psoriasis specialty clinic at Hope Hospital. 27% with severe psoriasis. Gender: 53% M, 47% F. Age: 35–51 years. 60 healthy control participants. Published COPE scores from other medical diseases.	PASI. PDI. COPE	Across medical conditions, patients showed remarkable similarity in the type of coping strategies used. The most frequently used coping strategies were acceptance, planning, active coping and positive reinterpretation. Patients with psoriasis were using social support strategies less frequently than controls.	Illness brings with it a generic form of coping that requires shaping to fit the specific demands of specific illnesses like psoriasis. Many of the difficulties experienced by patients with psoriasis are so demanding that they outstrip the coping resources of the patient, the family or social network. More research is needed on how to make coping more efficacious and particularly on how the family may be assisted in this respect.

[40]	Fortune et al., 2002b. United Kingdom. Psychology. Cross-sectional.	To investigate whether coping and alexithymia should be given significant consideration in the conceptualization of patient's adjustment to psoriasis, or whether the cognitive model (illness perception) of the condition held by patients is adequate on its own.	225 patients from Dermatology Centre at Hope Hospital, Salford. Gender: 52% M, 48% F. Age: 18–75 years. Mean age: 43 years.	PASI PLSI PDI PSWQ HADS IPQ Alexithymia Scale COPE	Cognitive psychological factors, mainly illness perception and to a lesser extent coping, appear to be more important for the patients' quality of life and psychological well-being than clinical severity.	The study emphasizes the importance of recognizing that the onset of a chronic condition brings with it a range of difficulties that may vary considerably in their nature and severity as perceived by the patient. Importance of cognitive factors represents a move towards more multidisciplinary model of patient care.

[41]	Richards et al., 2004. United Kingdom. Psychology. Cross-sectional.	To investigate the representation of psoriasis symptoms in psoriasis patients and their healthy partners, and to examine how their differences in beliefs about psoriasis may be associated with adaptive outcome in terms of anxiety, depression, and worry.	58 patients with chronic plaque psoriasis and their partners, recruited either from specialty clinic, dermatology outpatient clinic or inpatient ward setting. 49% M, 51% F. Mean age: 44 years; Mean duration of psoriasis: 18 years.	SAPASI IPQ-R HADS PSWQ	Patients with psoriasis had significantly higher levels of anxiety, depression and worry compared to their partners. The study indicated that divergence in patients' and partners' beliefs about emotional impact of psoriasis and chronicity of timeline accounted for statistical variance in depression for partners.	The study offers an insight into the way in which divergence, in terms of the way psoriasis is perceived by patients and their partners, may have an impact on psychological and clinical outcome. Moreover, the results illustrate the importance of concordance between the patients' and partners' models of psoriasis in relation to adjustment, and highlight the need to consider and collaborate with both patients and their partners in managing the challenging condition.

[42]	Jankowiak et al., 2004. Poland. Nursing. Cross-sectional.	To determine the need for health education among patients with psoriasis through the determination of gaps that most frequently occur in this kind of knowledge.	149 patients. Gender: 45% M, 55% F. Age: 13–78 years; mean age 41 years.	Questionnaire based on the PDI.	The study illustrated, among other things: (i) 54% reported stress as a cause while 43% were unable to report any factor causing the disease.	Patients with psoriasis need to expand their knowledge about the disease and self-care methods. The largest knowledge deficit observed were the factors causing the disease. There is a need for individualized health education programmes.

[43]	O'Leary et al., 2004. United Kingdom. Psychology. Cross-sectional.	To assess causal beliefs in a psoriasis population and to examine the link between these attributions and mood, quality of life, and health status. To measure perceived stress and examine the relationship between psychological well-being and psoriasis severity.	40 patients from an outpatient skin clinic and 101 from the Psoriasis Association. Gender: 58 M and 83 F. Mean age: 45 years (females were significantly younger than males). Mean duration of disease: 23 years.	PSS PDI SAPASI IPQ-R HADS	The most commonly endorsed causes were “stress/worry”. More than 60% of the sample scored above the scale midpoint for “stress/psychological” indicating a strong causal belief in this factor Levels of perceived stress, whilst strongly associated with mood and quality of life, were not associated with psoriasis severity.	A large proportion of people with psoriasis believe that stress is a causal factor in their illness. This belief was associated with poorer psychological well-being and the perception that psoriasis has a large emotional impact. Patients with psoriasis may need cognitive-behavioural interventions, which would allow individuals to identify their beliefs about their psoriasis and their medication and the impact that these have on their subsequent well-being and behaviour.

[44]	Fortune et al., 2004a. United Kingdom. Psychology. Prospective.	To investigate whether, and to what extent, improvement in the clinical severity of psoriasis induced by photochemotheraphy with psoralen plus ultraviolet A translates into meaningful changes in beliefs about psoriasis, coping, stress or disability.	72 patients with psoriasis referred for PUVA treatment. Gender: 45 M, 27 F. Mean age: 42 years; duration of psoriasis: 2–50 years. Response rate: 100.	PASI PLSI PDI PSWQ HADS IPQ COPE Scale	The study reported that the medical management of psoriasis has demonstrable effects on disability and psoriasis-related stress experienced by the current sample of patients, but not on levels of distress, beliefs about psoriasis or on the coping strategies used by patients.	The findings imply that clinical clearance of psoriasis is not sufficient to bring about changes in patients' distress. Unlike disability, distress in patients with psoriasis is possibly linked to the cognitive aspects of a patients' world view rather than to its more behavioural aspects. To minimize psychological distress and physical severity of the disease, patients with psoriasis may need multidisciplinary treatment programmes to challenge and change unhelpful beliefs about the condition, to develop and foster more appropriate coping responses.

[45]	Evers et al., 2005. The Netherlands. Psychology. Cross-sectional.	To examine whether generic physical, psychological and social factors relevant to patients with chronic diseases contribute to psychological distress in adults with psoriasis and atopic dermatitis.	128 patients with psoriasis and 128 patients with atopic dermatitis (aged over 16 years) from a dermatology clinic at University Medical Center, St. Radbound. Gender: 39% M, 61% F. Mean age: 48 years; mean duration of disease: 18 years.	Skin status assessed with a nine-item scale. Itching: four-item scale and VAS. Fatigue: VAS. IRGL. Disease Impact Scale. Illness Cognition Questionnaire. IRGL—Social Functioning Scale.	The study reported that higher levels of psychological distress were significantly related to physical symptoms of fatigue, a greater impact of disease on daily life, illness cognition of greater helplessness and less acceptance, less perceived support and a smaller social network.	The study indicates that at least 30% of the patients suffer from a higher level of psychological distress. Higher levels of fatigue, illness cognitions of greater helplessness and less perceived support significantly contribute to distress in these patients. The study indicates that patients with psoriasis could possibly benefit from multidisciplinary treatment options that focus on fatigue reduction (focusing on sleep disturbance, rest-activity balance), changing patients' pessimistic and helpless attitudes towards their disease by improving patients self-efficacy in coping with disease, and mobilizing social support from significant others.

[46]	Wahl et al., 2006. Norway. Nursing Cross-sectional.	To characterize how hospitalized patients coped with psoriasis and eczema during exacerbation of the disease in the period prior to admission to the dermatology ward, and to investigate the relationship between coping and quality of life.	146 hospitalized patients with psoriasis Gender: 69 M, 76 F. Mean age: 49.5 years. Mean duration of psoriasis: 19.1 years.	JCS. DLQI-N. The Perception of Living with Disease.	The results indicate that optimistic, belief-in-oneself and confrontational strategies are most frequently used among patients who are admitted to the dermatological ward for treatment. Use of confronting strategies is related to better quality of life. Duration of illness is significantly related to supportive coping strategy and belief-in-oneself. The longer one had the disease, the more often one used the belief-in-oneself mode. Women and younger people use emotional coping strategies more often than men and older people.	It appears that illness results in a generic form of coping that may require shaping to fit the individual demands of diseases like psoriasis, and that patients with psoriasis tend to use significantly less active coping strategies. Furthermore it seems that patients who have lived and functioned longer with psoriasis may have acquired more individual knowledge concerning the illness, thereby making them less dependent on support from others to cope with problems related to the disease. The findings highlight the complex features of the patients' psychological experiences of psoriasis and underline the need for integrating psychological interventions into standard care protocols.

[47]	Unaeze et al., 2006. USA. Medicine. Prospective.	To examine changes in specific aspects in which psoriasis may impact individuals over time, and to determine sociodemographic and clinical characteristics associated with HRQOL at baseline 1993 and with change in HRQOL over time.	867 patients completed questionnaire in 1993. 484 patients completed questionnaire in 2004 Gender: 62% M, 38% F. Mean age: 53 years (2004).	IPSO.	Responses to items assessing the impact of psoriasis on social aspects of HRQOL such as social activities, holiday or travel plans, and making new friends were generally stable. Over a period of more than a decade, impairment owing to psoriasis-related physical appearance decreased significantly (e.g., embarrassment/shame, unattractiveness, feeling like an outcast, which is among the worst aspects of psoriasis for the majority of patients).	The overall psychosocial impact of psoriasis on patients HRQOL decrease over time. The results suggest that chronic skin disease may become less burdensome compared to other health problems that increase with age.

[48]	Dubertret et al., 2006. Europe. Medicine. Cross-sectional.	To explore the patients' perspective of psoriasis on their lifestyle and well-being and to gain insight into effectiveness and satisfaction with current available therapies for psoriasis.	18.386 patient association members in seven European countries (Belgium, the Czech republic, Finland, France, Germany, Italy, and the Netherlands). Gender: 49% M, 51% F. Mean age: 30 years; Mean duration of psoriasis: 23 years. Response rate: 36.	Self-administered questionnaire developed in collaboration with EUROPSO (European Federation of Psoriasis Patients Associations) and the NPF (National Psoriasis Foundation). PDI.	(i) More than 70% reported psoriasis having an overall impact on their lives. The impact was greater with involvement of feet, armpits, genitals and hands. (ii) 48% of reported disability was accounted for by problems related to activities of daily living, especially relating to washing and changing clothes, the need to bathe more frequently, sporting activities, and problems with sleep. (iii) 50% report the fact that therapy is time-consuming as the most troublesome aspect of treatment, followed by ineffectiveness.	It is evident from this survey that patients with psoriasis suffer from significant impairment of their QoL. The perceived severity of disease is associated to the involvement of body area. It seems that self-reported severity of disease is associated with the overall impact of disease. The study enhances the understanding of complex interrelations between QoL impairment, psychological stress, disease severity assessment and patients' educational needs regarding their disease.

[49]	Uttjek et al., 2007. Sweden. Medicine. Qualitative.	The main issue is to find out how psoriasis affects the individual's everyday life, and if there is any variation between genders.	18 patients were selected from a population of a previous study with regard to gender, place of residence, and in relation to three district health care centres in Västerbotten. Age: 37–74 year, mean 58 years	Transcribed interviews.	The following themes emerged: (i) Marked by Visibility (ii) Adjustment (iii) Routinization (iv) Quality of life The most distinctive feature among both men and women, and with no variation between genders, was the feeling of being marked by the visibility of psoriasis in different social situations. Coping strategies did not differ among gender. The participants lived their lives with psoriasis in acceptance and/or restrictions, turning it into a routine in everyday life, which influenced the quality of life. Acceptance as well as routinization of the marking process developed with age, whereas concealing and avoiding were strategies used at all ages.	The worst situations occurred when patients were young and after onset of psoriasis, and included the visibility, the feeling of being marked, the struggle to conceal the disease and the fear of being rejected. Being with family or friends or being of some use contributed to good quality of life. The burden of being marked by the visibility was like an ever-present shadow for most of the patients and lead to restrictions in everyday life. Adolescence was found to be a tough period with psoriasis. As long as marking and discretion processes are going on in society, it is important to provide persons with psoriasis adequate help and support to not restrict their everyday life.

[50]	Eghlileb et al., 2007. United Kingdom. Medicine. Qualitative.	To identify the various ways in which the lives of relatives and partners of people with psoriasis are affected by the disease.	63 patients with psoriasis and their relatives or partners from a dermatology outpatients department of the University Hospital of Wales. Gender: 40 F 23 M. Age: 20–80 years. Mean age: 51 years.	Transcribed interviews and an open-ended postal questionnaire.	The impairment of relatives' lives were assigned to six different domains: (i) Treatment (ii) Psychological impact (iii) Social disruption (iv) Sport and leisure limitations (v) Daily activities (vi) Personal relationships	Psoriasis not only interferes with the daily lives and social functioning of patients with psoriasis, but also has a major impact on QoL of their relatives and partners. The study indicates the need to develop appropriate new care strategies for patients with psoriasis, also including their partners and families.

[51]	Watson and de Bruin 2007. South Africa. Psychology. Qualitative. Disease-specific factors. Self-experience burden. Personal factors.	To describe the lived experiences of men and women with psoriasis and how their perceived experiences impact the various dimensions of their self-concept.	7 patients with psoriasis. Gender: 3 M, 4 F. Age: 29–65 years. Illness duration: 11–60 years.	Patients' detailed descriptions of their perceptions and descriptions of their intrapersonal and interpersonal self as a person living with psoriasis.	The findings are synthesized in themes shared by all participants: (i) Impact of psoriasis on self-concept evaluation. (ii) Implications of treatment on the self-concept experience. (iii) Coping methods to enhance the self-concept. (iv) Meaning-making of the psoriasis self-concept experience. Psoriasis is described as invasive, intrusive, violating and disgusting, and is defined as an assault on the physical self that was unsightly, painful, embarrassing and humiliating. Psychological adapting to psoriasis was described as a journey of emotional and physical turmoil in the reconstructing of self.	Evident from the study is the tremendous power yielded by psoriasis in the arena of self-concept change. According to the participants, psoriasis is life changing radically, altering the self and the participants' being in contact with the self and others. Psoriasis knows no boundaries in its assault on the physical body. The experience of living with psoriasis leaves the patient feeling entrapped within an unfamiliar body Being young and single with psoriasis is described as devastating.

[52]	Amatya and Nordlind 2008. Sweden. Medicine. Qualitative.	To assess patients' perspective of pruritus in psoriasis vulgaris of plaque type.	20 patients selected from outpatient clinical records of the Department of Dermatology, Karolinska University Hospital. Intensity of itch > 4 by VAS. Gender: 7 M, 13 F. Age: 30–55 years. Mean disease duration: 17.7 years.	Transcribed interviews.	Pruritus is a common phenomenon in patients with psoriasis. Patients regard themselves as able to discriminate between pain and pruritus. Itch rather felt like pins and needles, and is characterized as burning and irresistible. Unbearable pruritus affects quality of life, that is, not taking part in general social activities and feelings of depression.	The study indicated that pruritus may be severe and affect quality of life in psoriasis patients.

[53]	Magin et al., 2009. Australia. Medicine. Qualitative.	To investigate the psychological co-morbidities in psoriasis in patients from general (family) practice and specialist practices.	29 patients with various disease duration and disease severity. Gender: 11 M, 18 F. Age: 25–71 years.	Transcribed interviews.	A schema of the interactions of psoriasis and psychological co- morbidities is developed with individual themes: (i) Appearance and self-image, self-esteem and self-identity. Subjects often expressed a belief that they were defined by their skin and reported diminished self-esteem. (ii) Behavioural consequences: Social interactions and lifestyle were circumscribed by avoiding activities leading to embarrassment and shame, caused by a sense that others were scrutinizing and judging character or intrinsic world.	Psychological sequelae are complex and encompass a range of psychological morbidities. Symptoms of anxiety and depression were often causally linked by respondents to experiences of embarrassment or shame or to the socially limiting effects of behavioural avoidance. This was overt in cases where children with psoriasis were teased or taunted. The study indicates that psychological sequelae are more common in females, but males are also markedly affected including concerns regarding appearance and social and sexual attractiveness. Patients with psoriasis need social support and a patient-centered approach to management, emphasizing coping strategies, patient education and subsequent involvement in management decisions might reduce psychological co-morbidity.

[54]	Globe et al., 2009. USA. Medicine. Qualitative.	To explore psoriasis patients' perception of the impact of psoriasis.	39 patients identified by general practitioners participated in 5 separate concept elicitation focus groups. 4 groups included 31 patients with severe psoriasis, and 1 group included 8 patients with mild psoriasis. Gender: 17 M, 22 F.	Transcribed interviews.	All participants reported itch as an important impact on everyday life. The affective dimensions of itch were described as unbearable, worrisome, bothersome and annoying. The sensory dimension of itch was described as burning, stinging, and crawling like ants. Itch symptoms affected sleep quality, concentration and regular physical activity. Some patients reported missing work or school because of itch symptoms.	From the patients' perspective, itch is one of the most important symptoms of psoriasis contributing to diminished health-related quality of life in patients with both mild and severe disease. The sensory dimension of itch is a significant predictor of depression, distress and sleep impairment. There is a need for assessment of itch in clinical practice to help patients with this troublesome symptom.

[55]	Ljosaa et al., 2010. Norway. Nursing. Cross-sectional.	To investigate (i) prevalence and characteristics of psoriasis-related skin pain and discomfort, (ii) evaluate differences in demographic/clinical characteristics among patients with or without skin symptoms, and (iii) to explore symptoms characteristics.	139 patients recruited prior to a consultation at the inpatient and outpatient dermatology units at a university hospital in Oslo. Gender: 44% M, 56% F. Mean age: 51 years.	Co-morbidity (SCQ-18). Prevalence of skin pain and skin discomfort. BPI. PQAS. PASI.	The study showed that (i) 41.7% of patients reported skin pain. (ii) 36.7% of patients reported skin discomfort. Significantly higher percentages of patients with pain (88–94%) reported that their symptoms interfered with mood, work, sleep, and relations with other people compared to patients with discomfort. Patients with pain reported significantly higher severity on items like enjoyment of life and daily activities.	Findings from this study suggest that psoriasis-related skin pain and skin discomfort may be a larger problem than previously estimated.

**Table 3:** Themes, clusters and verification of findings.

Theme	Cluster	Verification
Visibility of disease	Disease-specific factors	[37, 38, 45, 47–49, 51–54]
Onset time		
Fluctuating nature of disease		
Physical symptoms: itch, pain, discomfort	Self-experienced burden of disease (physical)	[37, 38, 40, 41, 44, 45, 47–55]
Bodily suffering		
Disease impact on self-concept	Self-experienced burden of disease (psychological)	
Psychological impact of disease		
Disease as a psychological burden		
Disease impact on family and partner	Self-experienced burden of disease (social)	
Disease impact on daily life		
Personal coping strategies	Personal factors	[37–46, 49, 51, 53]
Personal factors, illness perception		
Personal factors, partner, family and network		
